# Efficacy of tepotinib in patients with high-grade glioma with *MET* alterations: A case series

**DOI:** 10.1093/nop/npaf130

**Published:** 2025-12-31

**Authors:** Andrew Rodriguez, Karan Dixit, Richard M O’Hara, Morana Vojnic

**Affiliations:** Department of Neurosurgery, University of Kansas Medical Center, Kansas City; Department of Neurology, Northwestern University Feinberg School of Medicine, Chicago; EMD Serono, Inc., Boston, MA, United States, an affiliate of Merck KGaA; Department of Neuro-Oncology, Rutgers Cancer Institute of New Jersey, New Brunswick

**Keywords:** Glioblastoma, *MET* alterations, MET inhibitors

## Abstract

**Background:**

Glioblastoma is the most common and aggressive adult brain malignancy, characterized by poor prognosis and limited treatment options. The *MET* (hepatocyte growth factor receptor) oncogene plays a crucial role in tumor progression, with alterations such as gene amplification and fusion events contributing to worsened prognosis, decreased survival rates, and resistance to standard chemotherapy treatments.

**Case Summary:**

This case series documents the clinical outcomes of 5 patients with *MET*-altered glioblastoma treated with tepotinib, a highly selective MET tyrosine kinase inhibitor approved for patients with *MET* exon 14 skipping advanced/metastatic non-small cell lung cancer, in routine clinical practice in the United States. The patients received tepotinib through ad hoc off-label requests, with a daily dosage of 450 mg after progression following radiotherapy-based first-line treatment, with or without temozolomide. The efficacy and safety outcomes were assessed based on physicians’ evaluations. Tepotinib demonstrated clinical benefit, with partial response observed in 2 patients, stable disease in 2 patients, and progressive disease in 1 patient. The duration of tepotinib treatment ranged from 0.7 to >15 months, with treatment ongoing in 1 patient as of June 2025. Adverse events were generally mild to moderate, including grade 2 peripheral edema, grade 1 increased alanine aminotransferase levels, and grade 2 elevated liver function tests.

**Conclusions:**

These findings support the potential of tepotinib as a targeted therapy for *MET*-altered glioblastoma, consistent with preclinical evidence and previous case reports. Further research is warranted to better understand the efficacy and safety of MET inhibition in this patient population.

Glioblastoma is the most common and aggressive adult brain malignancy and is defined by the 2021 WHO classification of tumors of the central nervous system as a grade 4 isocitrate dehydrogenase (IDH)-wildtype diffuse adult-type glioma,^1^ for which *O^6^-methylguanine-DNA methyltransferase* (MGMT) promoter methylation status is prognostic.^2^ Despite improvements in survival rates for other brain tumors over the past decade, glioblastoma continues to have low survival rates, with a 5-year relative survival of 7%.^3^^,^^4^  *MET* (hepatocyte growth factor receptor) alterations are rare mutations in high-grade glioma, which may be associated with worsened prognosis, decreased survival rates, and resistance to standard chemotherapy treatments.^5^ Amplifications in *MET* are observed in approximately 2%-5% of glioblastoma cases, although higher rates of overexpression (13%-45%) have been noted in various studies.^5^^,^^6^  *MET* fusions are rare but can occasionally be seen in IDH-mutant astrocytomas, previously classified as secondary glioblastomas.^7^^,^^8^ Recurrent *PTPRZ1–MET* fusion has been reported in approximately 15% of secondary glioblastomas, although it is rare (0.4%) in primary glioblastomas.^7^^,^^9^ Chromosomal translocations involving *MET*, such as *PTPRZ1–MET*, lead to constitutive activation of MET and are associated with aggressive tumor behavior and poor prognosis.^5^^,^^10^ Advances in next-generation sequencing (NGS) techniques now allow for the identification of *MET* alterations in glioblastoma, facilitating more personalized treatment strategies.^11–13^ However, *MET* alteration testing is not universally recommended or a mandated diagnostic test for glioblastoma patients but is dependent on the clinician’s familiarity with emerging data on MET inhibitors.^5^ In *MET*-altered glioblastoma, aberrant signaling arising from *MET* amplification and *PTPRZ1–MET* fusions drives ligand-independent activation and upregulation of the PI3K/AKT, RAS/ERK, and STAT signaling pathways.^5^^,^^14^ Selective MET inhibitors like tepotinib (approved for patients with *MET* exon 14 skipping non-small cell lung cancer [NSCLC])^15^ block the MET receptor’s ATP binding pocket, preventing tyrosine autophosphorylation (Y1234/Y1235) and disrupting downstream pathways (PI3K/AKT, RAS/ERK, and STAT) that promote cancer cell growth and survival.^5^^,^^14^ In addition, tepotinib has been reported to cross the blood–brain barrier efficiently, reaching concentrations in the brain 2.87 times higher than in plasma. The unbound brain-to-plasma ratio observed was 0.25, suggesting adequate brain penetration for intracranial target inhibition with tepotinib.^16^ Indeed, patients with *MET* exon 14 skipping NSCLC who have brain tumors as target lesions evaluable by Response Assessment in Neuro-Oncology Brain Metastases criteria demonstrated an intracranial response rate of 66.7% with tepotinib.^17^ Therefore, MET inhibitors, such as tepotinib, may be effective in patients with *MET*-altered glioblastoma.^5^^,^^14^

Preclinical studies indicate that MET-targeted therapies, including monoclonal antibodies and small-molecule inhibitors like crizotinib, tepotinib, and capmatinib, can suppress tumor growth and improve treatment outcomes in *MET*-altered glioblastoma.^5^^,^^9^^,^^16^ Early-phase clinical trials of MET inhibitors have demonstrated promising results in improving overall survival for patients with recurrent glioblastoma harboring *MET* alterations.^5^^,^^18–20^ A recent case report of a patient in his late 20s with glioblastoma with leptomeningeal dissemination and *PTPRZ1–MET* fusion highlighted a prolonged complete response lasting 35 months to adjuvant tepotinib treatment.^21^ Here, we report a case series of 5 patients with *MET*-altered glioblastoma who received tepotinib in clinical practice in the United States.

## Methods

Patients received tepotinib 450 mg once daily (QD), provided by the manufacturer EMD Serono Research & Development Institute, Inc., Billerica, MA, United States, an affiliate of Merck KGaA, through ad hoc off-label requests, outside of a clinical trial or investigator-sponsored study setting in the United States. The efficacy and safety outcomes are reported based on physicians’ assessments, without the application of standardization criteria.

## Case Series

### Case 1

**Figure 1.**
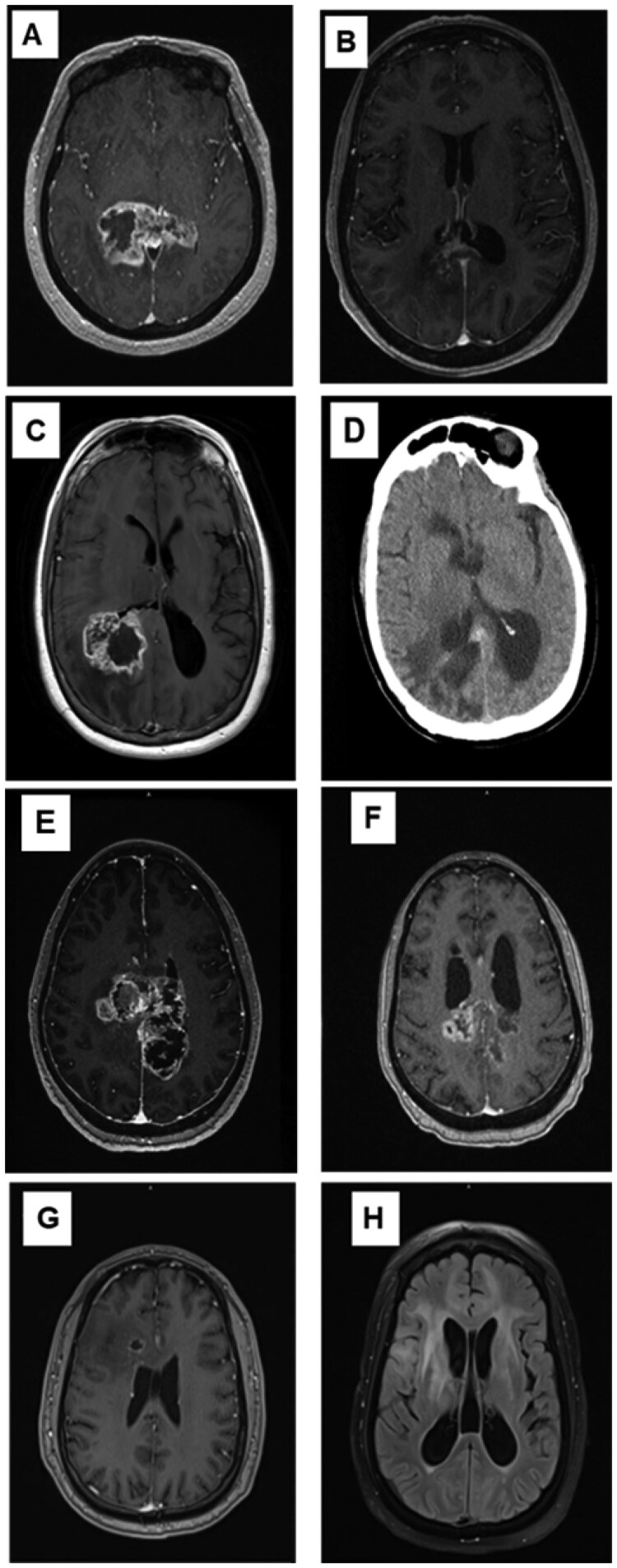
MRI of patients 1, 3, 4, and 5 at baseline and after tepotinib initiation. (A) Patient 1 scan at baseline (December 11, 2023). (B) Patient 1 scan at the latest follow-up post-tepotinib initiation (June 27, 2025). (C) Patient 3 scan at baseline (August 31, 2023). (D) Patient 3 scan after 3.7 months of tepotinib (June 2, 2024). (E) Patient 4 scan at baseline (January 23, 2024). (F) Patient 4 scan 3 weeks after tepotinib initiation. (G) Patient 5 scan at baseline (February 26, 2023). (H) Patient 5 post-contrast and T2 FLAIR images after 0.7 months of tepotinib treatment (September 25, 2023). MRI, magnetic resonance image.

In December 2023, a female in her 50s with a medical history of hypertension, hyperlipidemia, and glaucoma presented with headaches and visual changes. A brain magnetic resonance image (MRI) revealed an enhancing splenial mass. A stereotactic needle biopsy confirmed IDH-wildtype and MGMT-methylated glioblastoma, and NGS results identified *MET* amplification (copy number [CN] 33.9). Standard-of-care treatment with concurrent chemotherapy with temozolomide (75 mg/m²) and radiation (60 Gy in 30 fractions) was started. However, during the first 3 weeks of treatment, it was discontinued because of adverse effects associated with temozolomide. In March 2024, a post-radiation brain MRI demonstrated a splenial enhancing mass of similar size to the pre-radiation scan, with extensive worsening vasogenic edema and midline shift. At this stage, high-dose dexamethasone for cerebral edema and targeted therapy with tepotinib were recommended. The patient initiated tepotinib 450 mg QD in March 2024 and, as of June 2025, has been receiving it for more than 15 months with no adverse effects or laboratory abnormalities. Stable neurological findings, including partial field restriction, fatigue, and headaches (Karnofsky Performance Status [KPS] 80%-90%), have been reported, most of which were ongoing prior to tepotinib initiation. At the latest follow-up, more than 15 months after therapy initiation, brain MRI demonstrated an ongoing decrease in tumor burden and cerebral edema (Figure 1;  Figure S1), consistent with a partial response (PR), and KPS was 90%.

### Case 2

In January 2023, a female in her 30s presented with progressive headaches over the past month. A head CT scan without contrast showed large areas of vasogenic edema within the bilateral cerebrum, and an MRI revealed multifocal enhancing lesions throughout both cerebral hemispheres. The patient underwent a subtotal resection of the left frontal tumor. Later, histology, immunohistochemistry, and comprehensive molecular profiling confirmed the presence of diffuse pediatric-type high-grade glioma, H3-wildtype and IDH-wildtype, MGMT-unmethylated, WHO grade 4, with *PTPRZ1-MET* fusion. Treatment with whole-brain radiotherapy (WBRT) commenced at the end of January 2023. However, a week later, an MRI revealed tumor progression with an interval increase in right-sided cerebral edema. Targeted therapy with tepotinib 450 mg QD was initiated in May 2023 (KPS 60%). Treatment with tepotinib was continued for 11 months and was generally well tolerated, with the exception of grade 2 peripheral edema, which was managed through treatment interruption for 1 week and then using furosemide for 1 week. The best response achieved was stable disease (SD). The patient died 12 months after therapy initiation due to bacteremia.

### Case 3

In August 2023, a female in her 60s with a history of breast cancer presented with several weeks of left-sided neglect. MRI revealed a right parietal enhancing mass. Later, NGS testing of a biopsy confirmed glioblastoma, which was IDH-wildtype, MGMT-methylated, and *MET*-amplified (CN not available). The patient received 6 weeks of chemoradiotherapy with concurrent temozolomide. From November 2023 to February 2024, the patient had several hospitalizations related to worsening gait, failure to thrive, deep vein thrombosis, and pulmonary embolism, which precluded the use of adjuvant chemotherapy. Imaging revealed communicating hydrocephalus and a slight increase in the size of the right parietal necrotic brain mass. Serial high-volume lumbar punctures showed elevated protein levels and negative malignant cytology. At this time, targeted therapy with tepotinib (450 mg QD) was initiated due to the presence of *MET* amplification (KPS 50%). Approximately 2 months after treatment initiation, brain MRI findings were most consistent with post-radiation changes rather than tumor progression. However, 3.6 months after tepotinib initiation, the patient presented to the hospital following a seizure. CT imaging revealed a small intracerebral hemorrhage, although post-radiation findings remained stable (Figure S2). No dose modifications, lab abnormalities, or adverse events (AEs) with tepotinib were reported. Due to gradual progressive cognitive deficits, the patient moved to hospice care 3.7 months after tepotinib initiation, at which time tepotinib was discontinued, with a best response of SD (KPS 40%). The patient died 5.9 months after therapy initiation.

### Case 4

In January 2024, a female in her 50s presented with several weeks of right leg weakness, short-term memory loss, word-finding difficulty, and behavioral changes. Brain MRI showed a large, necrotic pericallosal mass with leptomeningeal enhancement, and preliminary brain biopsy pathology was consistent with high-grade glioma (Figure S3). High-dose steroids and palliative WBRT (30 Gy in 10 fractions) were administered at the start of February. Final pathology with NGS testing confirmed glioblastoma, which was IDH-wildtype, MGMT-indeterminate, with a *PTPRZ1–MET* fusion. In late February 2024, tepotinib 450 mg QD was initiated (KPS 40%). Three weeks after tepotinib initiation, a brain MRI demonstrated a significant decrease in tumor size and an improvement in leptomeningeal enhancement (Figure 1; Figure S3). At this time, mental status improved toward baseline with a reduction in encephalopathy. Tepotinib was continued and tolerated well without any serious AEs. After 1.5 months of receiving tepotinib, the patient developed asymptomatic grade 1 alanine aminotransferase (ALT) elevation, which improved spontaneously without requiring a dose adjustment (KPS 60%). However, 2.6 months after tepotinib initiation, the patient exhibited progressive lower extremity weakness, encephalopathy, and bowel/bladder incontinence. Imaging findings on a brain MRI were concerning for disease progression, and the patient transitioned to hospice care. Tepotinib was discontinued after approximately 2.8 months with a best response of PR (KPS 40%); the patient died 4.5 months after tepotinib initiation.

### Case 5

In February 2023, a male in his 40s with a history of hypertension presented with unresponsiveness at home. A brain MRI revealed an enhancing mass in the right frontal lobe with ependymal enhancement of the right lateral ventricle, and a biopsy identified high-grade glioma. Pathological and NGS analysis confirmed glioblastoma, which was IDH-wildtype, MGMT-unmethylated, with *MET* amplification (CN not available). Initially, treatment with concurrent temozolomide (75 mg/m^2^ daily) and radiation therapy (60 Gy in 30 fractions) was given for 6 weeks. In July 2023, follow-up MRI scans revealed an increase in right frontal lobe enhancement and ventriculomegaly, suggestive of tumor progression with leptomeningeal dissemination. Adjuvant temozolomide therapy (150 mg/m^2^, 5/28-day cycle), in combination with bevacizumab (10 mg/kg every 2 weeks), was administered for 2 months. In late August 2023, the patient was hospitalized for progressive lethargy and fatigue over a 3-week period (KPS 70%). Cerebrospinal fluid findings suggested disseminated glioblastoma with leptomeningeal involvement, and therefore, high-dose dexamethasone was started. In September 2023, targeted therapy with tepotinib (450 mg QD) was initiated based on the presence of *MET* amplification; however, the patient was discharged to inpatient hospice care 3 days later due to progressive neurological decline (KPS 40%). The patient’s mental status, communication, and nutritional intake improved while continuing tepotinib. After 1 week, the patient was transferred back to the hospital for ongoing oncological care (KPS 60% on readmission). Asymptomatic grade 2 elevated liver function tests (LFTs) were reported, which resolved after dose reduction of tepotinib to 225 mg QD. Six days after admission, the patient experienced seizures and worsening mental status. A brain MRI showed disease progression, leading to hospice care, where the patient died in October 2023 (Figure S4). Tepotinib had been received for a duration of approximately 0.7 months, with a best response of progressive disease (PD).

## Discussion

This case series reports clinical experience with tepotinib in patients with *MET*-altered glioblastoma (mostly female, aged 30-70 years), including those with advanced disease such as leptomeningeal dissemination. Overall, tepotinib showed clinical benefit with PR in 2, SD in 2, and PD in 1 patient (Table 1). Treatment durations ranged from 0.7 to over 15 months, with 1 patient still on therapy as of March 2025.

**Table 1.** Summary of case series

Patient	Sex	Age,^a^ years	IDH wildtype	MGMT methylation status	*MET* alterations	Prior therapy	LOT, tepotinib^b^	Best response, tepotinib	DOT, tepotinib	AEs, tepotinib	AE management
1	F	50-60	Yes	Positive	*MET* amplification	Temozolomide (75 mg/m^2^) + radiotherapy (60 Gy in 30 fractions)	2nd	PR	15 months (ongoing)	None	None
2	F	30-40	Yes	Negative	*PTPRZ1–MET* fusion	Radiotherapy (45 Gy in 25 fractions [with sequential boost to 54 Gy]) + dexamethasone (6 mg every 6 h)	2nd	SD	11 months	Grade 2 peripheral edema	Compression stockings and massage
3	F	60-70	Yes	Positive	*MET* amplification	Temozolomide + radiotherapy (60 Gy in 30 fractions)	2nd	SD	3.7 months	None	None
4	F	50-60	Yes	NA	*PTPRZ1–MET* fusion	Radiotherapy (30 Gy in 10 fractions)	2nd	PR	2.8 months	Grade 1 elevated ALT	None
5	M	40-50	Yes	Negative	*MET* amplification	1) Temozolomide (75 mg/m^2^ daily) + radiotherapy (60 Gy in 30 fractions) 2) Temozolomide (150 mg/m^2^) + bevacizumab (10 mg/kg every 2 weeks for 2 months)	3rd	PD	0.7 months	Grade 2 elevated LFTs	Dose reduction (225 mg QD)

a Approximate age of the patient at presentation.

b 450 mg QD.

Abbreviations: AE, adverse event; ALT, alanine aminotransferase; DOT, duration of treatment; IDH, isocitrate dehydrogenase; LFT, liver function tests; LOT, line of therapy; *MET*, hepatocyte growth factor receptor gene; MGMT, O^6^-methylguanine-DNA-methyltransferase; NA, not applicable; PD, progressive disease; PR, partial response; QD, once daily; SD, stable disease.

Tepotinib demonstrated a manageable safety profile with grade 2 peripheral edema, grade 1 increased ALT levels, and grade 2 elevated LFTs observed in 1 patient each, while 2 patients were reported not to have any tepotinib-related AEs. These results indicate the real-world clinical effectiveness of tepotinib in patients with glioblastoma with either *MET* amplification or *PTPRZ1–MET* fusion glioblastoma, consistent with preclinical evidence,^9^ previous case reports,^21^ and observations with other MET inhibitors.^22^^,^^23^

Standard treatment for glioblastoma involves surgical resection followed by concurrent radiation and chemotherapy; however, this approach has failed to improve the prognosis for patients with glioblastoma.^24^ In contrast, MET-targeted therapies have demonstrated potential in preclinical studies and early clinical trials.^9^^,^^25^ For instance, subgroup analysis of a phase II clinical trial with MET inhibitor onartuzumab + bevacizumab in recurrent glioblastoma patients with high hepatocyte growth factor (HGF) expression had improved progression-free survival and overall survival compared with patients with low HGF expression.^25^ However, only 5 of 129 patients had *MET*-altered tumors, making meaningful analysis difficult.^25^ Given the rarity of *MET* alterations in this disease setting, conducting clinical trials may require national or large-scale initiatives. Indeed, challenges include suboptimal patient selection, difficulties in accurately identifying driver mutations, and the limited ability of investigational drugs, which cross the blood–brain barrier. Also, incorporating *MET* alteration testing as part of the initial tumor profiling and diagnosis, utilizing NGS and molecular profiling techniques, will improve patient selection and outcomes.

The limitations of this case series include its real-world context, potential bias in the cases reported, and a lack of standardized response criteria or safety reporting. Additionally, obtaining insurance approval for tepotinib to be used for an off-label use can be particularly challenging due to the absence of randomized trial data in this patient subpopulation. Another limitation of the use of tepotinib in this setting is the development of potential resistance mechanisms in *MET*-altered gliomas, specifically involving the epidermal growth factor receptor and phosphatidylinositol-4,5-bisphosphate 3-kinase catalytic subunit alpha pathways; however, this has not been investigated here. Lastly, we could not rule out radionecrosis as part of the initial radiographic/clinical course in patients 1 and 2.

## Conclusion

In conclusion, this case series demonstrates that tepotinib may provide meaningful clinical benefit with manageable AEs in US patients with glioblastoma harboring either *MET* amplification or a *PTPRZ1–MET* fusion mutation in clinical practice.

## Supplementary Material

npaf130_Supplementary_Data

## Data Availability

Not applicable as this is a case series.
