# Supplementary material for: Efficacy of tepotinib in patients with high-grade glioma with MET alterations: A case series
Source: Neurooncol Pract. 2025 Dec 31;13(2):422–7. doi: 10.1093/nop/npaf130 (PMC13153682; doi:10.1093/nop/npaf130)

# Supplementary material

**Supplementary Figure 1.** MRI of Patient 1 over time. (A) Scan at baseline (December 11, 2023). (B) Scan after radiotherapy, prior to tepotinib initiation (March 15, 2024). (C) Scan at first follow-up post-tepotinib initiation (April 28, 2024). (D) Scan at follow-up post-tepotinib initiation on October 20, 2024. (E) Scan at follow-up post-tepotinib initiation on March 18, 2024. (F) Scan at the latest follow-up post-tepotinib initiation (June 27, 2025).


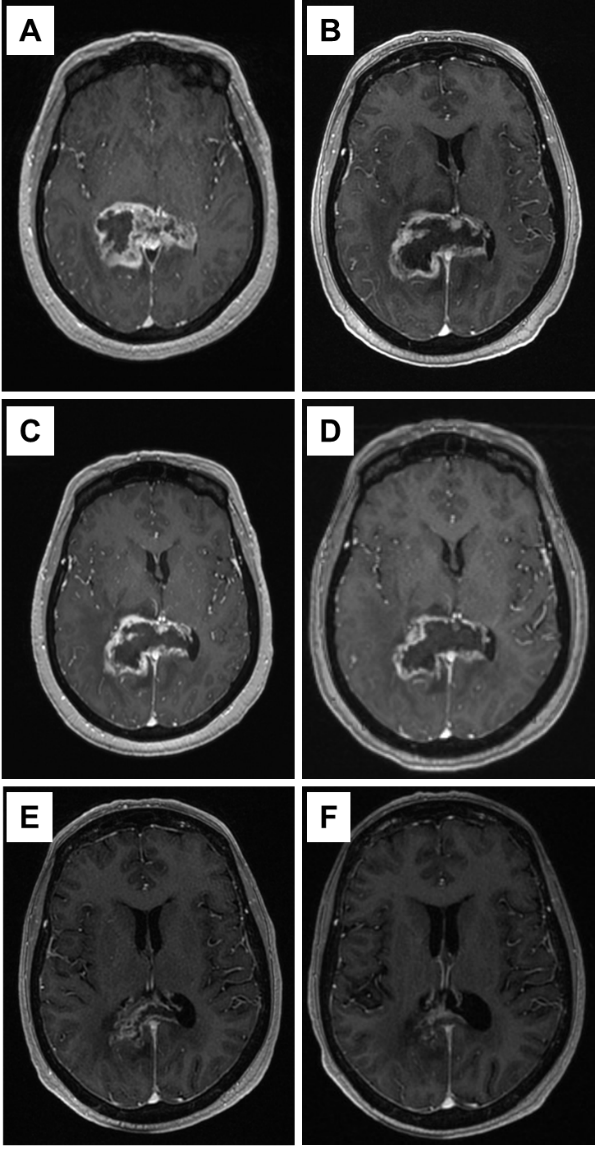


**Supplementary Figure 2**. MRI of Patient 3 over time. (A) Scan at baseline (August 31, 2023). (B) Scan after resection and chemoradiotherapy (February 2, 2024). (C) Scan after tepotinib initiation (April 12, 2024). (D) Scan after 3.7 months of tepotinib (June 2, 2024).


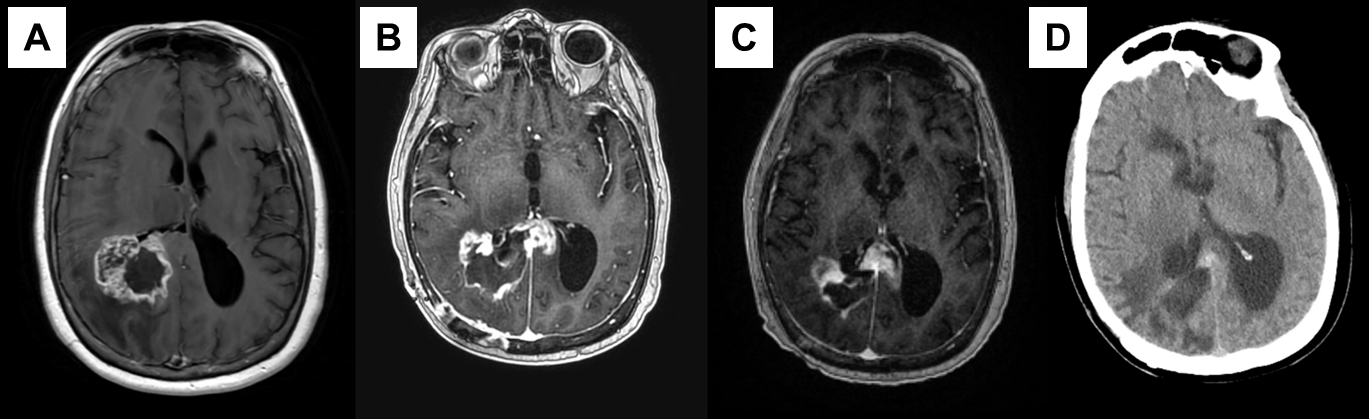


**Supplementary Figure 3**. MRI of Patient 4 over time. (A–D) Scans at baseline (January 23, 2024). (E–H) Scans 3 weeks after tepotinib initiation (March 18, 2024).


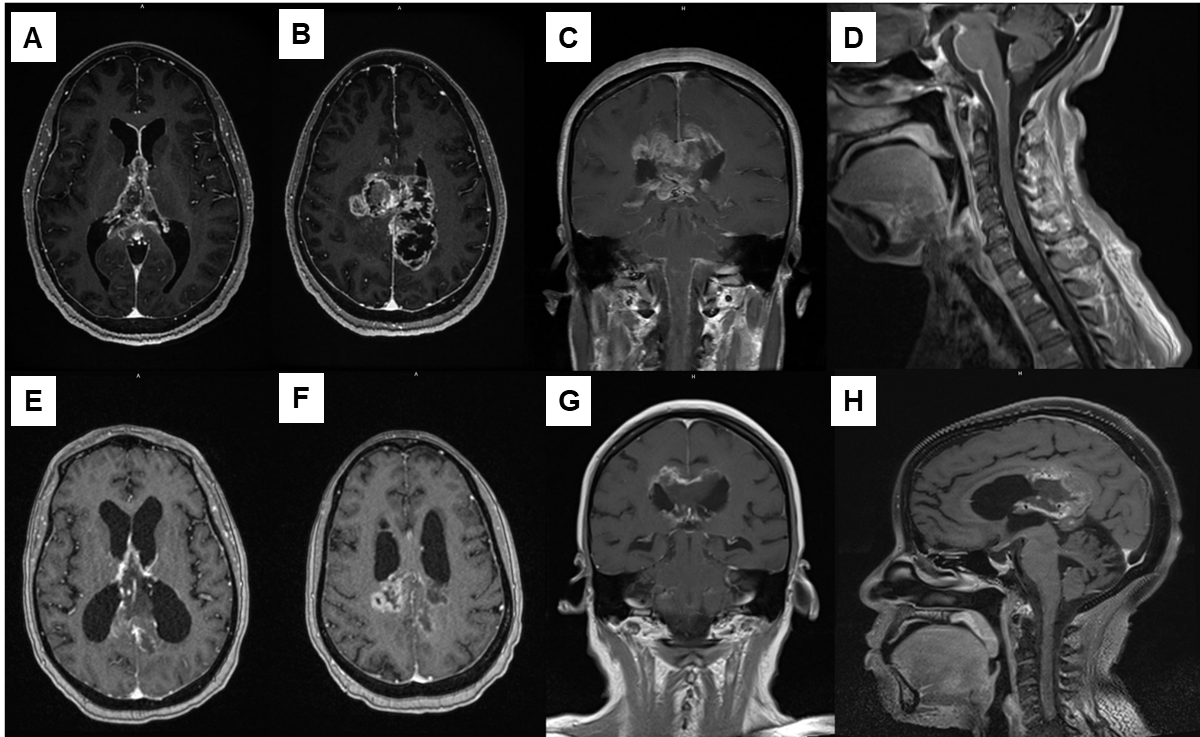


**Supplementary Figure 4**. MRI of Patient 5 over time. (A) Scan at baseline (February 26, 2023). (B) Scan after resection and chemoradiotherapy (July 5, 2023). (C) Scan at tepotinib initiation (August 25, 2023). (D–E) Post-contrast and T2 FLAIR images after 0.7 months of tepotinib treatment (September 25, 2023).


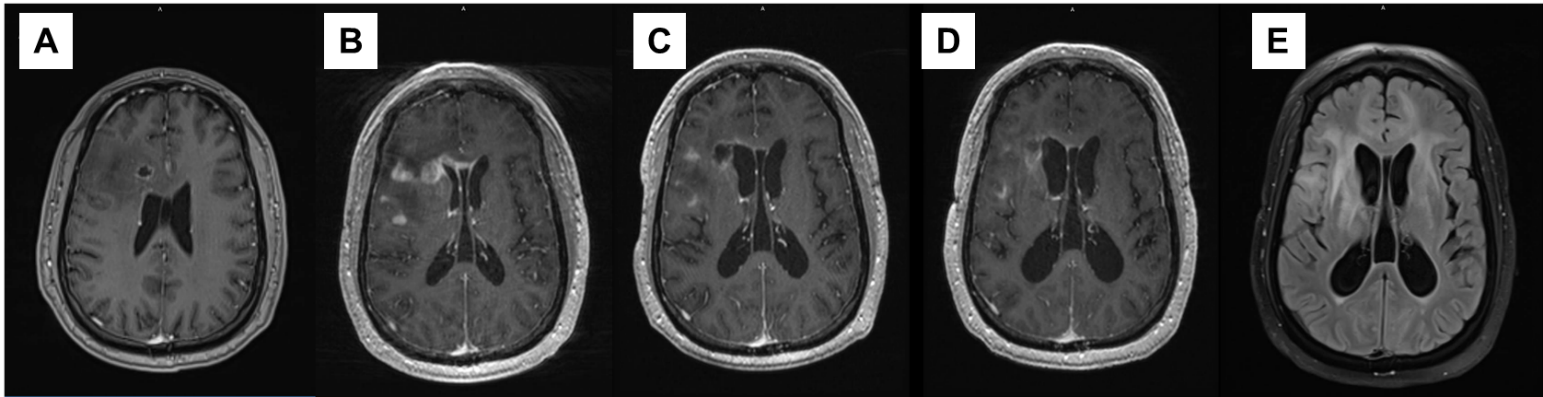

Supplement: npaf130_Supplementary_Data [file npaf130_supplementary_data.docx]
